# *FGF14*‐related episodic ataxia: delineating the phenotype of Episodic Ataxia type 9

**DOI:** 10.1002/acn3.51005

**Published:** 2020-03-12

**Authors:** Julie Piarroux, Florence Riant, Véronique Humbertclaude, Ganaelle Remerand, Jessica Hadjadj, Franck Rejou, Christine Coubes, Lucile Pinson, Pierre Meyer, Agathe Roubertie

**Affiliations:** ^1^ Département de Neuropédiatrie CHU Gui de Chauliac Montpellier France; ^2^ Service de Génétique Moléculaire Neurovasculaire Groupe hospitalier Saint‐Louis ‐ Lariboisière ‐ Fernand Widal AP‐HP Paris France; ^3^ Service de Médecine Psychologique Enfants et Adolescents CHU Saint Eloi Montpellier France; ^4^ Service de néonatologie CHU Estaing Clermont‐Ferrand France; ^5^ Service de Génétique Clinique Département de Génétique Médicale Maladies Rares et Médecine Personnalisée Hôpital Arnaud de Villeneuve CHU de Montpellier Montpellier France; ^6^ PhyMedExp U1046 INSERM UMR9214 CNRS Montpellier France; ^7^ INSERM U 1051 Institut des Neurosciences de Montpellier Montpellier France

## Abstract

We report four patients from two families who presented attacks of childhood‐onset episodic ataxia associated with pathogenic mutations in the *FGF14* gene. Attacks were triggered by fever, lasted several days, and had variable frequencies. Nystagmus and/or postural tremor and/or learning disabilities were noticed in individuals harboring *FGF14* mutation with or without episodic ataxia. These cases and literature data delineate the *FGF14‐*mutation‐related episodic ataxia phenotype: wide range of age at onset (from childhood to adulthood), variable durations and frequencies, triggering factors including fever, and association to chronic symptoms. We propose to add *FGF14*‐related episodic ataxia to the list of primary episodic ataxia as Episodic Ataxia type 9.

## Introduction

Hereditary cerebellar ataxias constitute a large, heterogeneous group of neurological diseases presenting as a cerebellar syndrome, variably combining gait alteration, limb incoordination, dysarthria, and eye movement abnormalities. *FGF14* mutations have been identified as a rare cause of autosomal dominant spinocerebellar ataxia type 27 (SCA27). SCA27 is characterized by gait and limb ataxia, dysarthria, and nystagmus; tremor is often associated and may be the presenting manifestation. Other symptoms, including orofacial dyskinesias, psychiatric manifestations, cognitive delay, or parkinsonism are common. Symptom onset occurs early in life nevertheless the disease course is usually very slow and motor function is maintained through life in most of the patients.^1^ More recently, animal model studies and genome‐wide association studies suggested that FGF14 may be a risk factor for various neuropsychiatric diseases including depression addiction and schizophrenia, as well as neurodegenerative diseases,^2^, ^3^ *FGF14* encodes fibroblast growth factor 14 highly expressed in the brain and especially in Purkinje cells, where it interacts with voltage‐gated Na+ channels to regulate neuronal excitability. FGF14 also regulates synaptic transmission from granule cells to Purkinje cells and plays a role in synaptic plasticity and neurogenesis in the hippocampus.^3^, ^4^ The occurrence of episodic ataxia (EA) has been occasionally associated with *FGF14* mutations.^5^, ^6^, ^7^, ^8^, ^9^ Here, we report four patients from two families harboring *FGF14* mutations with a peculiar phenotype including the variable combination of fever‐triggered EA and permanent mild neurological symptoms.

## Material and Methods

### Standard protocol approvals, registrations and patient consents

The study was carried out in accordance with the Declaration of Helsinki and was approved by the local ethical committee. Written informed consent was obtained from the patient's legal representatives.

### Genetic analysis

Patient IV‐10 underwent Sanger sequencing for *CACNA1A*, *KCN1A*, *PRRT2,* and *FGF14*. Index case from family B was analyzed using a panel of genes known to be associated with episodes of ataxia (*CACNA1A*, *KCNA1*, *CACNB4*, *SLC1A3*, *FGF14*, *SLC2A1*, *ATP1A3*, *PRRT2*). Sanger sequencing was used to confirm the variant in this patient and to test the relatives of patient IV‐10.

## Results

### Family A

**Figure 1:**
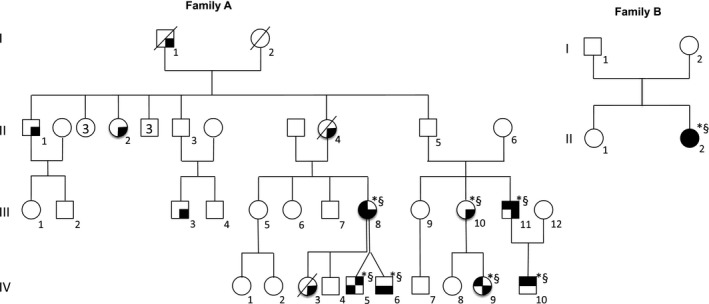
Pedigrees of families A and B. Black upper right quadrant: episodic ataxia; black upper left quadrant: nystagmus; black lower right quadrant: tremor; black lower left quadrant: developmental delay/learning difficulties; *: mutation carrier; §: clinical examination performed by a neurologist, a neuro pediatrician or a geneticist.

**Patient IV‐10** was the index case of a large family previously briefly published (Fig. 1A).^10^ Pregnancy and delivery were uneventful. Psychomotor achievements were normal (head control acquired at 3 months, independent walking at 15 months, first sentences at 18 months). He was diagnosed with hyperopia at 20 months.

At 24 months, a multidirectional nystagmus was noticed. Shortly after, the patient exhibited a first episode of vertigo with nausea, vomiting, and unsteady gait during a febrile illness; it lasted two days then completely resolved. Attacks of ataxia during fever recurred three to four times a year. The last episode occurred at age 4, consisting of severe ataxia with an inability to walk and dizziness for almost a week. Between the attacks, clinical examination only disclosed multidirectional nystagmus without a tremor. At age 5, clinical examination, academic achievements and cognitive assessment were normal.

**Patient III‐11** was patient IV‐10's father. At 8 years he exhibited an episode of vertigo with inability to walk and dizziness, lasting one week and triggered by a febrile illness; the second episode of gait unsteadiness occurred at 31 years during a period of intense stress. Nystagmus was diagnosed in infancy. He reported having tremor of the upper limbs (UL) from childhood, without functional impairment, and was never referred for this symptom. He succeeded in university studies. Clinical examination at 37 years disclosed horizontal nystagmus and mild UL rest tremor.

**Patient IV‐5** was the second cousin of patient IV‐10. He was born at 36 weeks of gestation, from a dizygotic twin pregnancy. Birth parameters were normal. At 3 months, he exhibited two accesses of breath‐holding spells. He experienced a first episode of acute ataxia at 2.8 years, after a febrile illness; it lasted 2 days, and then the patient regained prior neurological function. After this event, the mother noticed monthly paroxysmal attacks of gait disorders. They were never observed by a physician, they were described by the mother as gait unsteadiness with bizarre posture of the left limb, triggered by fatigue or intercurrent illness, lasting several hours and resolving spontaneously. These episodes did not recur after the age of 5.5. The patient walked unaided at 14 months; language delay and behavioral disturbances (poor attention, sleep difficulties) were noticed from the age of 36 months. Clinical examination at 5 years disclosed very discrete action tremor. At last follow‐up (6.5 years), neurological examination showed coordination disabilities without tremor. The boy exhibited gross and fine motor skills difficulties (unable to get dressed or cut his food alone, unable to ride a bicycle, very poor graphism abilities). He could associate a few words but not form full sentences. He benefited from language and psychomotor rehabilitation from age 3. Due to learning difficulties, he underwent a special educational program.

**Patient IV‐6** was the twin brother of patient IV‐5. Birth parameters were normal. He managed to walk alone at 14 months; language was delayed. At 6.5 years of age, he exhibited gross and fine motor skills difficulties, discrete UL postural tremor with coordination difficulties, language disabilities related to expressive dysphasia, poor attention span, but displayed no ataxia and was less severely affected than his brother. He also benefited from language and psychomotor rehabilitation and special educational program. He never experienced paroxysmal neurological episode.

Their mother (**Patient III‐8)** reported UL tremor from childhood without functional impairment, and she never sought medical advice concerning this tremor. She never exhibited paroxysmal neurological episode. She experienced learning disabilities and reached the middle school level. Clinical examination at 45 years disclosed mild symmetric postural tremor and nystagmus.

**Patient IV‐9** had a normal psychomotor development. Minutes‐lasting episodes of spontaneously resolving lower limb hypertonia were reported between 7 and 9 months of age. Nystagmus was reported from the first months of life, and UL postural tremor was noticed during the second year, associated with fine motor skills disturbances.

Her mother **(Patient III‐10)** exhibited UL postural tremor from childhood.

Genetic analysis identified the variant NM_004115.3:c.439G>T leading to a stop mutation p.Glu147Ter in *FGF14* gene in the index case and patients III‐11, IV‐5, IV‐6, III‐8, IV‐9, and III‐10. Other family members were reported having hand tremor from childhood, but genetic testing was not performed (Fig. 1A).

### Family B

**Patient II‐2** exhibited delayed motor and language skills (Fig. 1B). Vertical nystagmus was noticed at one year, and UL tremor was noticed soon after. At 2 years she exhibited a first access of severe ataxia with inability to walk and sit unaided, triggered by a febrile illness; after one week she regained her previous neurological status. Such episodes, always triggered by fever, recurred twice a year until the last follow‐up (6.3 years). Clinical examination then disclosed nystagmus, discrete UL postural and action tremor, and coordination difficulties. Mild learning difficulties were reported, and she benefited from language rehabilitation. She was described as very smiley and communicative with her peers.

Genetic analysis identified the variant NM_004115.3:c.486_487del leading to a premature stop codon p.Tyr162Ter in *FGF14* gene. Due to parental child neglect, she was placed in foster care from the age of 7 months, and the parents were not available for analysis.

## Discussion

We report four patients harboring a dominant *FGF14* mutation who presented prolonged episodes of seldom‐recurring, fever‐triggered EA with childhood onset (Table 1). EA are rare genetic disorders characterized by brief recurrent attacks of ataxia, sometimes associated with other ictal symptoms (tremor, dysarthria, vomiting, vertigo, headache) and typically a normal interictal neurological examination. Triggering factors in episodic ataxia include physical and psychological stress, startle, sudden movements, fatigue, caffeine, alcohol, or fever.^11^, ^12^, ^13^ Eight genetically distinct subtypes have been described (Table 2).

**Table 1:** Main clinical features of the patients harboring FGF14 mutation.

Family	Family A								Family B
Patient	IV‐10	III‐11	IV‐5	IV‐6	III‐8	IV‐9		III‐10	patient I‐2
Sex/age at last follow‐up (years)	M/5	M/37	M/6.5	M/6.5	F/45	F/3		F/NK	F/6.3
Acute episodes of ataxia	Yes	Yes	Yes	No	No	No		No	Yes
Age at onset (years)	2	8	2.8						2
Triggering factor	Febrile illness	Febrile illness, stress	Febrile illness						Febrile illness
Duration	2–7 days	7 days	2 days						7 days
Total number	8 No recurrence after age 4	2	1						Twice a year until last follo‐up
Symptoms	Vertigo, ataxia, nausea, vomiting, occacional confusion	Dizziness, ataxia	Ataxia						Severe ataxia
Other paroxysmal signs	No	No	2 access of breath holding spell at 3 months; between 2.8 and 5.5 years, daily access of gait unsteadiness with bizarre posture of the left limb lasting several hours	No	No	Brief episodes of lower limb hypertonia lasting some minutes between 7 and 9 months of age		No	No
Permanent symptoms	Yes	Yes	Yes	Yes	Yes	Yes		Yes	Yes
Tremor (age at onset)	No	UL postural tremor (from childhood)	No	UL postural tremor (from childhood)	UL postural tremor (from childhood)	UL postural tremor (from 2.5 years)	UL postural tremor (from childhood)		UL postural tremor (from infancy)
Nystagmus (age at onset)	Yes (from 24 months)	Yes (from infancy)	No	No	Yes	Yes (from infancy)		No	Yes (from infancy)
Permanent ataxia	No	No	No	No	No	No		No	No
Learning difficulties	No	No	Yes	Yes	Yes	No		NK	Yes
Psychomotor development	Normal	Normal	Language delay	Language delay	Normal	Normal		NK	Global delay
Paraclinical investigations (age)	Blood electrolytes, lactate, aminoacid, ammonemia, acylcarnitine dosage, urinary organic acid screening, cerebrospinal fluid analysis, auditory and vestibular assessment, brain MRI: normal (2 years)	None	Auditory and vestibular assessment, CGH‐array, FMR1 gene analysis, brain MRI normal (3.3 years)	None	None	None		None	EEG, brain MRI, ophthalmological assessment normal (1.2 years)

UL, upper limbs; M, male; F, female; NK, not known.

**Table 2:** Cardinal features of the various subtypes of EA. Clinical features of childhood‐onset EA related to FGF14 mutation are emphasized in bold characters.

EA type (gene)	EA1 (KCNA1)	EA2 (CACNA1A)	EA3 (12q42, no gene)	EA4 (No gene‐	EA5 (CACNB4)	EA6 (SLC1A3)	EA7 (No gene)	EA8 (UBR4)	EA9 (FGF14)
Age at onset (years)	Childhood	Childhood or adolescence	1–40	20–50	20–60	From infancy to adulthood	<20	Infancy	From **childhood** to adulthood
Attacks duration	Seconds to minutes	Hours to days	Minutes to hours	Brief	Hours	Hours to days	Hours to days	Minutes to hours	Seconds to **several days**
Triggering factors	Movement, stress, fatigue, caffeine, alcohol	Exertion, fatigue, stress	Stress, fatigue, sudden changes in head position	Changes in head position, fatigue, environmental motion		Fever, stress, heat, smoking	Excitement, exercise	Anxiety, cold, stress	**Fever**, stress, physical activity
Interictal manifestations	Myokymia	Nystagmus	Myokymia	Nystag‐mus, abnormal smooth pursuit	Nystag‐mus, ataxia	Nystagmus, ataxia	None	Nystag‐mus, ataxia, myoky‐mia, tremor	Nystagmus, upper limb postural and action tremor
Additional features/atypical manifestations	Epileptic seizures, myokimia, prolonged attacks,rigidity and cataplexy, deafness, distal weakness	Other forms of episodic neurological syndroms, permanent ataxia,developmental delay,cerebellar atrophy	Epileptic seizures	Epileptic seizures	None	Cognitive deficit,hemiplegic migraine	None	None	**Develop‐mental delay, Learning disabilitiesP Paroxysmal dyskinesia**
Follow‐up	Improve‐ment with age	Improvement after acetozo‐lamide	Poor therapeutic response	Poor therapeutic response	Poor therapeutic response	Variable response to acetazo‐lamide	Poor therapeutic response	Poor therapeutic response	Variable **Improvement with age**

NK, not known.

EA associated with *FGF14* mutations have been previously reported in 8 patients from 5 families^5^, ^6^, ^7^, ^8^, ^9^, ^14^ (Table 3). Literature analysis and the precise clinical description of our four patients allow better delineation of the phenotype of EA related to autosomal dominant *FGF14* mutations: (i) wide range of age at onset (early childhood such as in our patients, to adulthood), (ii) variable triggering factors, especially fever (7/10 patients with available data), (iii) variable duration with long‐lasting attacks not uncommon (often mimicking febrile cerebellitis), (iv) variable frequency (ranging from limited number of episodes with spontaneous improvement before the teens to weekly access), (v) associated to chronic upper limb tremor and/or nystagmus, (vi) pharmacological responsiveness still unknown (improvement after acetazolamide treatment in 2 patients).^9^ (vii) genetic heterogeneity including point mutations and gene deletions. The *FGF14* mutation was also identified in family members exhibiting a milder phenotype (isolated nystagmus or upper limb postural tremor with childhood‐onset, without EA). Patients IV‐5 and IV‐9 exhibited paroxysmal manifestations suggesting paroxysmal dyskinesia, as previously described in one case^15^; some of these episodes spontaneously resolved in the first years of life, which is unusual compared to other forms of PD.^13^

**Table 3:** Main clinical features of patients with paroxysmal neurological manifestations (episodic ataxia, paroxysmal dyskinesia) harboring FGF14 mutation previously reported in the literature.

Patient	Choquet Patient 1	Choquet Patient 2	Choquet Patient 3		Amado Patient 1 & 2	Choi	Coebergh patient 1	Schesny	Shimojima
Chromosomal abnormality/*FGF14* gene mutation	NM_004115:c.211_212insA/p.(Ile71Asnfs*27)				Deletion of 424 kb on chromosome 13q33.1 including *FGF14*	NM_175929:c.31A>/p.(Thr11Ala)	Deletion of 202 kb on chromosome 13q33.1 including exons 1‐4 of *FGF14*	NM_175929:c.208+1G>A	Translocation t(13;21)(q32;q22.3) disrupting *FGF14*
Sex/age at last follow‐up (years)	M/31	M/NK		F/NK	F/NK	M/46	M/6	M/twenties	M/6
Acute episodes	Episodic ataxia	Episodic ataxia		Episodic ataxia	Episodic ataxia	Episodic ataxia	Episodic ataxia	Episodic ataxia	Paroxysmal dyskinesia
Age at onset	26 years	NK		NK	Childhood	39 years	Childhood	Twenties	8 months
Triggering factor	None	Fatigue exercise		NK	Fever	NK	Fever	High emotional stress levels, physical activity, certain body positions (e.g., bending forward), and caffeine intake	Crying
Duration	NK	20 min		NK	NK	Hours	NK	Min to hours	5 min
Frequency (or total number)	NK	4 times per week		Rare	NK	NK	(3 episodes)	4 times per month	Several per week
Symptoms	Incoor‐dination, unsteady gait, vertical oscillopsy, dysarthria, headache	Dysarthria, unsteady gait, and diplopia		Vertigo dysatrhia	Ataxia	Dizziness, headache	Ataxia	Intense vertigo, dizziness, nausea, dysphagia, diplopia	Attacks of choreic movements
Treatment	Acetazo‐lamide discontinued due to adverse effects	No		No	No	Response to acetazolamide	No	Improvement after acetazolamide	Valproic acid and phenobarbital non effective
Other paroxysmal signs	Attacks of right upper limb dystonia	No		Head‐ache	No	No	No	No	Breath holding spells from 9 months
Permanent symptoms	Yes	Yes		Yes	Yes	Yes	Yes	Yes	No
Tremor	Yes (From age 29)	No		No	Yes	No	No	UL postural tremor	
Nystagmus	Yes (From age 29)	Yes		Yes	Yes	Yes	Yes	Yes	
Ataxia	Yes (From age 29)	No		No	Yes	No	Yes (From age 2)	Slight	
Learning difficulties	NK	NK		NK	Yes	No	Yes	No	Yes (mental al disability)
Psycho‐motor develop‐ment	NK	NK		NK	NK	Normal	Delayed	Delayed	Mental deficiency

NK, not known; UL, upper limb.

FGF14 regulates the Cav2.1 presynaptic channels by modulating the current and the vesicular recycling.^4^ EA could result from this dysregulation, at least partially. FGF14 also directly regulates the function of Nav1.2 and Nav1.6 channels at the axon initial segment. NaV1.2 is the main channel subtype implicated in the mediation of fever‐induced neuronal hyperexcitability.^16^ This could explain why fever is a triggering factor in FGF14‐mutated patients.

Some genes involved in progressive ataxia may also be responsible for episodic neurological manifestations: recently, permanent or even progressive neurological signs including ataxia, combined with various types of other episodic syndromes have been reported in patients with the so‐called « episodic ataxia» (linked to *CACNA1A, PRRT2, SLC2A1*).^11^, ^13^, ^17^ FGF14‐associated phenotypes emphasize this overlap between progressive and episodic ataxias, with phenotypic continuum encompassing chronic (SCA27) and paroxysmal ataxia. Learning difficulties in most of our patients and in previously published cases strongly enlarge the implication of *FGF14* mutation in developmental disabilities^6^, ^7^, ^15^ and is concordant with the putative role of FGF14 in behavioral, cognitive, and psychiatric disturbances.^2^, ^3^

We propose that fever‐triggered EA associated with upper limb tremor in the patients and/or in relatives suggest *FGF14* involvement; genetic analysis (targeted gene or panel gene testing and if negative, gene deletion testing) establishes the diagnosis. We propose to add *FGF14*‐related EA to the list of primary EAs as “type 9 episodic ataxia.”

## Conflicts of Interest

The authors declare that they have no conflict of interest related to the research covered in the article.
